# Memorandum on the Training of the Territorial Medical Service

**Published:** 1909-06-12

**Authors:** 


### The Royal Army Medical Corps Section.

### MEMORANDUM ON THE TRAINING OF THE TERRITORIAL MEDICAL SERVICE.

An important memorandum has recently been issued by the Army Council indicating the lines on which the training of the different branches of the Territorial Medical Service should be conducted, and it is very satisfactory to note that it is made an opportunity for expressing high approval of the work done by the medical units during last year's training season, notwithstanding the many difficulties inseparable from the early stages of any large scheme of reorganisation.

Taking first the Medical Service with regimental units, the memorandum impresses on regimental surgeons that they are not relieved of the duty of caring for the sick of their units because a field ambulance is present in camp. This fact is not sufficiently recognised. A field ambulance goes to camp for combined training in field work with the brigade to which it may be attached, and if its work is to consist of looking after the sick in camp it will lose the only opportunity it has of making itself efficient for service in the field. Besides, to do so would take it out of its proper zone of work. It is a fundamental principle that a medical unit with a field army should never be depleted either in personnel or material for any outside duties.

In training camps during peace the memorandum lays it down that soldiers unfit to remain in their lines may be sent for a period of two days to the field medical unit, but not for longer; but it favours rather the formation of a specially organised camp hospital under the senior medical officer for the treatment and disposal of the sick and injured of the various units in camp. Such an arrangement recommends itself on every ground, and it supplies what has been found to be a deficiency in the present scheme of the Territorial Medical Service. A camp hospital of this kind would correspond to the "Clearing Hospitals" of the Regular Army, the need for which is now acknowledged, and it would give the field ambulances full opportunities for their field training. The establishment of these camp hospitals at once raises the question as to where their personnel is to be obtained. They cannot be staffed by fatigue duty men, who know nothing of nursing; nor are the regimental stretcher-bearers either qualified or available for the work. Men with a knowledge of hospital duties are needed, and some plan will have to be devised for furnishing these. They might be obtained from the certificated men of the Ambulance Corps of the St. John's and St. Andrew's Ambulance Associations in England and Scotland respectively, receiving pay while in camp, or they might be provided for by the enrolment in every unit of an ambulance squad of six men, who would be borne as supernumerary on the strength of the regiment and be specially trained by the regimental surgeon for the work. This latter plan has much to recommend it, as it would furnish a very valuable body of men with a knowledge of nursing available for staffing the clearing hospitals which the Territorial Force will require in time of war just as much as the regular army. We note with satisfaction that the memorandum announces that it is proposed to issue one medical companion and water-bottle and one surgical haversack and water-bottle to each headquarter and regimental unit, and we hope that funds will allow of this being done without any delay, as the absence of such equipment in the past has been in our opinion a grave defect.

Passing next to the subject of camp sanitation, the memorandum dwells on the great need for a preliminary training by lectures and demonstrations of the sanitary section of each unit before going to camp, where the teaching can be made, as it ought to be, in every way practical. What is said about the sanitary section applies equally to the water-duty men when they are provided and trained by the unit itself, but the intention at present seems to be to enrol these men as a separate body, attach them to the divisional school of instruction for training, and then distribute them to units when they go into their annual camp. Very properly the memorandum insists on the necessity of carefully considering the important questions of water-supply, refuse, and sewage disposal before a camp is occupied. Of this there can be no doubt, and as it is suggested that definite plans and instructions should invariably be drawn up on these points after the site has been visited and reported on by the divisional or other sanitary officer, we would further recommend that the prospective senior medical officer of the camp or another medical officer appointed by him should obtain permission from the A.M.O. to pay a preliminary visit of inspection to the camp some time before its occupation, so as to make himself acquainted with the water supply and to satisfy himself that it is protected from all danger of pollution in the arrangements for its conveyance and distribution to the troops.

Another procedure, too, that we think should always be followed is to arrange for a medical officer accompanying the fatigue party that goes in advance to prepare the camping ground for the main body. Unless this is done and proper precautions taken, the sanitary section of the unit may arrive to find the camping ground fouled and dirty, making it sometimes very difficult to have matters cleaned and put right. Especially is this the case if another regiment has occupied the ground previously, and has not left the camp properly cleaned. If this should happen, the presence of a medical officer with the advance party would be advantageous, as he could make a report on the exact conditions found, and thus help to put a stop to a form of carelessness that might have serious dangers on active service. Every outgoing regiment should leave its camping ground in a clean and sanitary condition.

Only a brief reference is made in the memorandum to the general hospitals. It is recommended that at present their personnel should not attend camp, as their training is best carried out in a military hospital. Probably the Herbert Hospital at Woolwich would furnish the best course of instruction in the very important duties these men have to discharge.

It is to the training of the field medical units that the memorandum devotes the greater part of its remarks, and there is laid down at once the excellent principle that a complete study of medical duties in the field is the main point to be kept in view. With this we are in thorough accord. In the past a great deal of useful time was wasted in combined training with other branches of the service before ever the men of the old bearer companies and field hospitals knew their respective duties. In view of the new constitution of the field ambulances, which combine the functions of the old bearer companies and field hospitals in the shape of their bearer and tent divisions, it is most essential that the personnel should have a thorough training in these dual duties, especially as the three sections into which a field ambulance can be split up have each their share of bearer and tent divisional work. To enable this to be done satisfactorily, the memorandum advises that not only should a unit train together as a whole, but normally all the field ambulances of one division should train in one camp at one time, and not necessarily, for the present, with other troops. It is unnecessary to give in detail all the suggestions contained in the memorandum as to the lines on which the instruction of the field ambulances should be carried on both as regards the preliminary work in the drill halls and afterwards in camp. Suffice it to say that they outline a very high standard of proficiency, but one we feel certain not beyond the intelligence and energy of the officers, non-commissioned officers, and men who now fill the ranks of the Territorial Medical Service, whose sole desire it is to be in every way efficient for the duties and stress of war.

